# Critical Predictors of the Outcomes of Rotator Cuff Arthroscopic Repair

**DOI:** 10.1055/s-0045-1802963

**Published:** 2025-04-28

**Authors:** Mauro Coura Perez, Cládis Lopes Sanches Filho, Pedro Cordeiro Moraes, Rodrigo de Almeida Chame de Castro, Gabriel de Araújo, Vinicius Schott Gameiro

**Affiliations:** 1 Divisão de Ortopedia, Rede Sarah Hospitais de Reabilitação, Brasília, DF, Brasil; 2 Departamento de Cirurgia Geral e Especializada, Faculdade de Medicina, Universidade Federal Fluminense, Niterói, RJ, Brasil

**Keywords:** arthroscopy, elderly, retrospective studies, rotator cuff injuries, shoulder pain

## Abstract

**Objective** To evaluate the functional outcomes after rotator cuff arthroscopic repair in an adult population and to establish risk factors for poor prognosis in this procedure.

**Methods** This retrospective cohort study analyzed medical records of 302 shoulders that underwent arthroscopy for rotator cuff repair between October 1, 2014, and December 31, 2017. Patients were included if they had a complete rotator cuff injury and met other specific criteria. Surgical procedures were performed consistently, and postoperative care involved a structured rehabilitation protocol. The primary outcome measure was the need for further surgery or a University of California, Los Angeles (UCLA) shoulder score < 28 at 2 years postsurgery. Statistical analyses included descriptive, inferential, and logistic regression methods.

**Results** Treatment success was observed in 234 shoulders (77.5%), while 68 shoulders (22.5%) were classified as treatment failures. Factors significantly associated with treatment failure included having more than 2 affected tendons (OR: 2.8, 95%CI: 1.5–5.0) and lesion sizes greater than 25 mm (OR: 2.9; 95%CI: 1.6–5.3). Patients aged 75 years or older had a failure rate of 54.5%, compared to 21.3% in younger patients ( *p* = 0.019). The Goutallier classification for fatty degeneration was significantly worse in older patients ( *p* = 0.001). Critical patients, defined as those aged ≥ 75 years with a Goutallier classification > 2 and lesion sizes > 25 mm, had a treatment failure rate of 75.0% (OR: 11.2; 95%CI: 2.2–57.0).

**Conclusion** Rotator cuff arthroscopy showed no worse prognosis based solely on age until 75 years. However, patients older than 75 years with significant muscle degeneration and large lesions had substantially increased chances of poor outcomes. Alternative treatments should be considered for these critical patients to improve their quality of life and upper limb functionality.

## Introduction

The increase in life expectancy and the desire to maintain healthy conditions in the labor market have prompted orthopedists to reconsider the conservative treatment of complete rotator cuff lesions in adults. Approximately 50% of patients over 70 years old have asymptomatic full-thickness rotator cuff tears, with about half becoming symptomatic within 2.8 years and seeking tendon repair to regain normal function. 1 Despite the established correlation between tendon degeneration, tear size, and patient age with postoperative outcomes, more comprehensive data is needed to guide treatment decisions effectively.

Rotator cuff injuries are a common cause of shoulder pain and disability, particularly among the elderly. Previous studies have shown that factors such as tear size, tendon degeneration, and patient age significantly influence the outcomes of rotator cuff repair surgeries. 2 3 4 Studies by Gasbarro et al. 4 and Wu et al. 2 have demonstrated that larger tears and greater tendon degeneration are associated with higher rates of surgical failure. Similarly, patient age has been identified as a critical factor, with older patients often experiencing poorer outcomes due to reduced healing capacity and increased comorbidities. 3

However, there is a lack of consensus on the specific age threshold beyond which the prognosis significantly worsens. Additionally, the combined effect of age, tear size, and tendon degeneration on functional outcomes has not been thoroughly investigated in a large cohort. This gap underscores the need for further research to identify critical predictors of surgical success and failure in rotator cuff repair.

The present study aims to evaluate the impact of age, tendon degeneration, and tear size on functional outcomes after rotator cuff arthroscopic repair. We were also able to determine a critical age threshold associated with a worse prognosis and identify key predictors of treatment failure in this patient population. By providing a nuanced understanding of these factors, we hope to assist surgeons in making more informed decisions regarding the suitability of rotator cuff repair for their patients.

## Materials and Methods

### Study Design

The current retrospective cohort study followed the Strengthening the Reporting of Observational studies in Epidemiology (STROBE) guidelines 5 and the Declaration of Helsinki. We analyzed our hospital medical records of all consecutive shoulder arthroscopy surgeries to repair complete rotator cuff injuries from October 1, 2014, to December 31, 2017, resulting in an eligible sample of 302 shoulders.

The inclusion criteria were all patients with rotator cuff injuries admitted for arthroscopy surgery for rotator cuff tendon repair. The exclusion criteria included patients younger than 18, those with unavailable clinical evaluations or imaging in medical records, individuals with previous shoulder surgery, those with irreparable cuff injuries, and patients whose contralateral shoulder was already included.

### Surgical Procedures

All surgeries were performed at a single hospital by the same two shoulder surgeons. Patients were positioned in a lateral decubitus position with the arm held by a traction device at 15 degrees of forward flexion and 45 degrees of abduction with up to 3 kg of traction.

Three portals (posterior, anterior, and lateral) were used to inspect the labrum, biceps tendon, and rotator cuff tendons. Lesions were repaired using 1 to 4 absorbable suture anchors until proper closure was achieved. The surgical technique included acromioplasty and, if necessary, biceps tenotomy or tenodesis. Specific repair techniques, such as single- or double-row sutures, were employed based on tendon quality and tear size.

### Postoperative Care

Postoperative care involved a sling, and abduction pads for 6 weeks.

Strengthening exercises began 12 weeks postsurgery. Patients were evaluated using clinical examinations and functional scales at regular intervals, with a final assessment at 2 years.

### Outcome Measures

The primary outcome measure was the need for further surgery or a University of California, Los Angeles (UCLA) shoulder score < 28 at 2 years post-surgery, indicating treatment failure. 6 Degeneration of cuff muscles was assessed using MRI and classified by the Goutallier classification. 7 8 Lesion sizes were measured intraoperatively with a 5-mm probe and categorized as smaller than 25 mm or 25 mm and larger. 9

### Statistical Analysis

We conducted descriptive and inferential analyses to profile patients in 2 groups based on prognosis (with and without failure) and UCLA scores before and 2 years after surgery. Variability in the quantitative variable distribution was considered low for coefficient of variation (CV) < 0.20, moderate for 0.20 ≤ CV < 0.40, and high for CV ≥ 0.40. Inferential statistical analysis evaluated the significance of differences in independent groups, UCLA scores pre- and postoperatively, and variable correlations.

To evaluate correlations between two qualitative variables, we used Chi-squared or Fisher test. If a significant correlation was found, the odds ratio (OR) estimated the risk of treatment failure, evaluated by the 95% confidence interval (95%CI).

For inferential analysis of quantitative variables, we compared distributions of quantitative or ordinal variables in two independent groups using the Mann-Whitney test. For comparisons involving more than two independent groups, the Kruskal-Wallis test was used. The Wilcoxon test compared UCLA scores before and after arthroscopy.

In the Wilcoxon signed-rank, the effect of the intervention received on the UCLA score was evaluated using Glass's Δ effect size measure: Δ in which *m*_1_ denotes the mean of the variable in the first evaluation (pre-intervention), *m*_2_ denotes the mean of the variable of the second evaluation (postintervention), and *s*_1_ is the standard deviation of the variable in the preintervention. This index is used in clinical trials 7 and comprises the sample value of the standardized mean difference through the standard deviation of the control group without failure, which more closely reflects the population's standard deviation. 10 11 12

The classification of Glass's Δ proposed by Rosenthal (10) was used as follows: insignificant: *Δ* ≤ 019; small: 020 ≤ *Δ* ≤ 049; medium: 050 ≤ *Δ* ≤ 079; large: 080 ≤ *Δ* ≤ 129; and very large: *Δ* ≥ 130. The effect size using Wilcoxon nonparametric tests ( *r* ) was also calculated by the quotient between the z-value (from the Mann–Whitney test) and the square root of the total sample number. The classification adopted for the effect size of non-parametric tests was the one proposed by Pallant: very small for *r* < 01; small for 01 < *r* ≤ 03; medium for 03 < *r* < 05; and great for *r* > 05. 13

The association between two quantitative variables was investigated using Spearman's rank correlation coefficient. Correlation was considered strong if the absolute value was greater than 0.7 and moderate if between 0.65 and 0.7. The Forward Wald method was used for variable selection in logistic regression analysis with a cutoff point equal to the proportion of failures in the sample. Model quality was assessed by the significance of the final model's parameters, OR, and measures of accuracy, sensitivity, and specificity. All significance tests were conducted with a maximum significance level of 5% ( *p* < 0.05), rejecting the null hypothesis if the *p* -value was less than 0.05. 11 14 15 16

## Results

The study initially included 332 shoulders. Based on the exclusion criteria, 6 shoulders with irreparable lesions identified during surgery, 5 shoulders with insufficient medical data, and 19 shoulders from the opposite side of patients already included were removed. Consequently, the results are based on an eligible sample of 302 shoulders that underwent arthroscopy for rotator cuff repair.

### Surgical Procedures Characteristics

In summary, patients undergoing arthroscopy for tendon suture typically had right-sided lesions (72.5%). The majority of lesions were degenerative (93.4%), and the “single-row” technique was used in 92.1% of cases. Lesion sizes were predominantly smaller than 25 cm (56.6%), and 2 tendons were most commonly affected (49.7%). In 52.0% of cases, there were no alterations in the long head of the biceps, while biceps tenodesis was performed in 37.7% of patients. The most common type of acromion was type II (49.7%), and the incidence of instability associated with tendon rupture was 20%.

### Treatment Outcomes

**Fig. 1:**
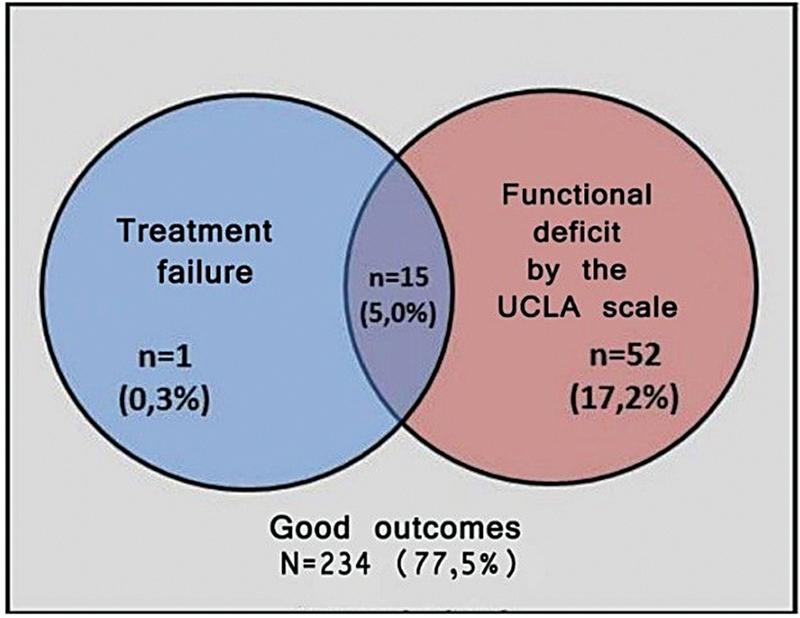
Distribution of cases by outcomes observed within 2 years after arthroscopy.

Treatment success was observed in 234 shoulders (77.5%), while 68 shoulders (22.5%) were classified as treatment failures. Treatment failure was defined by the need for further surgery or a UCLA shoulder score < 28 at 2 years postsurgery. Among the failures, 67 cases (22.2%) were due to a UCLA score indicating poor function, and 15 cases required additional surgery ( Fig. 1 ).

The Kruskal-Wallis test showed no significant difference between groups in pre and postoperative UCLA scores, nor in improvement between patients with an intact long head of the biceps or those who underwent tenodesis or tenotomy.

Improvement in UCLA scores was not correlated with acromion type or shoulder critical angle (SCA), indicating that these variables did not significantly affect outcomes.

### Influence of Tendon Involvement

When analyzing the frequency of the lesion characteristics of the patients in the sample, it was observed that the distribution of the number of affected tendons was significantly different in the groups with and without failure ( *p* = 0.009). When we dichotomized this variable into “more than 2 affected tendons” (yes or no), an association with a worse prognosis was evident among those who fit this classification (17.8% versus 37.5%; *p* < 0.001). The OR was 2.8 (95%CI: 1.5–5.0).

### Lesion Size

Lesions were categorized as < 25 mm or ≥ 25 mm.

In patients with lesions ≥ 25 mm, the OR for treatment failure was 2.9 (95%CI: 1.6–5.3), indicating a significant increase in poor outcomes.

We also examined the effect of lesion size and more than two affected tendons on prognosis. Our findings showed that 23.2% of cases had both more than 2 affected tendons and lesions ≥ 25 mm. Within the favorable prognosis group, only 18.4% had these combined characteristics. Conversely, in the poor prognosis group, 39.7% had both more than 2 affected tendons and lesions ≥ 25 mm ( *p* < 0.001). The OR for poor prognosis in patients with these combined factors was 2.9, indicating that these patients were nearly 3 times more likely to experience poor outcomes. This OR was significant, with a CI of 1.6 to 5.3, meaning the result is statistically robust and the CI does not include 1.

### Age as a Predictor

Age significantly predicted treatment outcomes. Patients younger than 75 years had a failure rate of 21.3%, while those aged 75 years or older had a failure rate of 54.5% ( *p* = 0.019). However, there was no significant difference in the improvement of UCLA shoulder scores between these age groups ( *p* = 0.068).

### Goutallier Classification

**Fig. 2:**
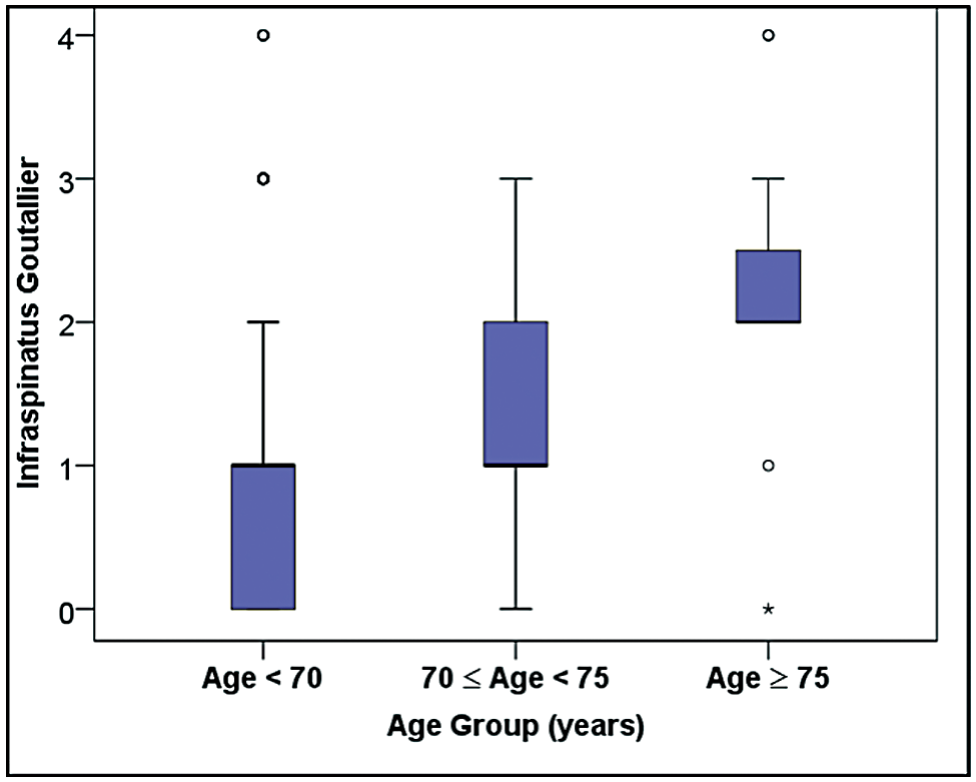
Boxplots of distributions of infraspinatus Goutallier classification according to age group.

The Goutallier classification for fatty degeneration of the infraspinatus muscle was significantly worse in patients aged 75 years or older compared to younger patients ( *p* = 0.001). This classification assessed the degree of muscle degeneration and its impact on surgical outcomes ( Table 1 and Fig. 2 ).

**Table 1:** Main nonparametric statistics of the Goutallier classification in the subgroups defined according to the age group for the cutoff points of 70 and 75 years

Goutallier classification	Age < 70 years ( *n* = 270)	Age ≥ 70 years and < 75 years ( *n* = 21)	Age ≥ 75 years ( *n* = 11)	Kruskal-Wallis *p* -value test
SUPRASPINATUS				
Minimal	0.0	0.0	1.0	**0.007**
Maximum	4.0	4.0	4.0	
Quartile 1	**1.0**	**1.0**	**2.0**	
Median	2.0	2.0	3.0	
Quartile 3	3.0	3.0	3.0	
**INFRASPINATUS**				**0.001**
Minimal	0.0	0.0	0.0	
Maximum	4.0	3.0	4.0	
Quartile 1	0.0	1.0	2.0	
Median	1.0	1.0	2.0	
Quartile 3	1.0	2.0	2.5	
**SUBSCAPULARIS**				0.189
Minimal	0.0	0.0	0.0	
Maximum	3.0	3.0	2.0	
Quartile 1	**0.0**	**0.0**	**0.5**	
Median	1.0	1.0	1.0	
Quartile 3	1.0	1.0	1.0	

### Critical Group Analysis

**Fig. 3:**
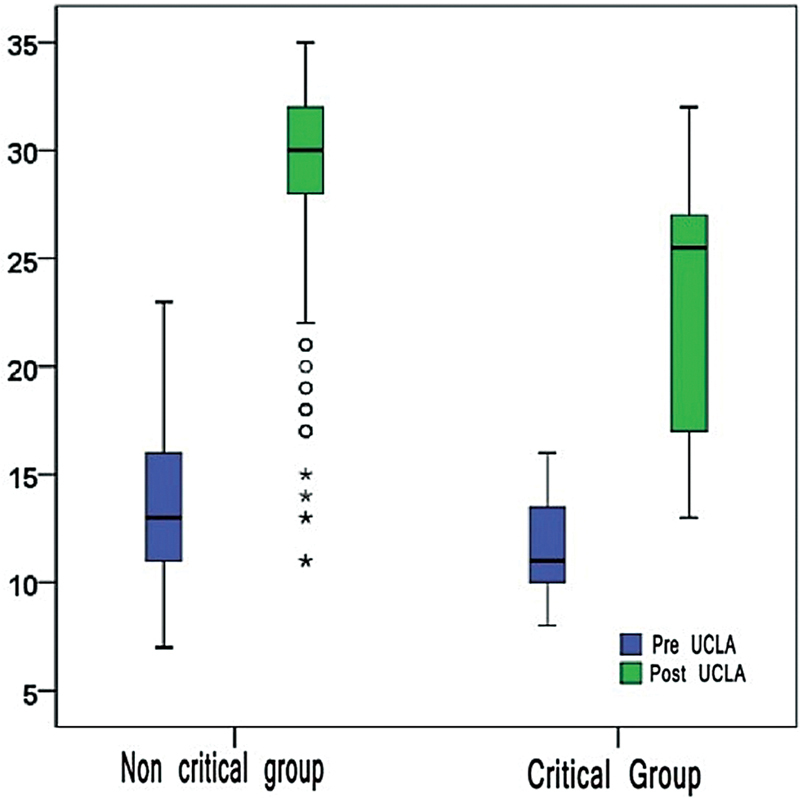
Boxplots of UCLA measurements before and after surgery in the critical and noncritical groups.

Critical patients, defined as those aged ≥ 75 years with a Goutallier classification of the infraspinatus muscle > 2 and lesion sizes ≥ 25 mm, represented 2.6% of the sample (8 patients). The incidence of poor prognosis in this group was 75.0% compared to 21.1% in non-critical patients ( *p* < 0.001), as shown in Fig. 3 . The OR for treatment failure in the critical group was 11.2 (95%CI: 2.2–57.0).

### Logistic Regression Analysis

All significant factors identified were proposed as predictors for failure in a multivariate logistic regression analysis. Table 2 shows the critical group results. The forward Wald method was employed for variable selection, using a cutoff point equal to the proportion of failures in the sample (0.23). The results of the final model are presented in Table 3 .

**Table 2:** Analysis of factors associated with failure

Factor	Fault				*p* -value*	OR (95%CI)
	No		Yes			
**Critical group = age > 75 years old + Goutalier IS >2 + lesion > 2.5 cm**						
No	232	78.9%	62	21.1%	< 0.001**	**11.2 (2,2–57.0)**
Yes	2	25.0%	6	75.0%		

**Abbreviations:** 95%CI, 95% confidence interval; IS, infraspinatus; OR, odds ratio.

**Notes:** *Chi-squared test. **Fisher's exact test was used.

**Table 3:** Logistic regression model for the occurrence of failure

Factor	Coefficient B	Standard error	Wald data	Degree of freedom	*p* -value	OR = Exp ( B)	lower limit of the 95% CI for Exp. (B)	95%CI for Exp (B) upper limit
Number of tendons > 2	1.075	0.296	13.193	1	0.0 00	2.92 9	1.64 0	5.230
Being of the critical group	1.896	0.854	4.926	1	0.0 26	6.66 0	1.24 8	35.531
Constant	-1.553	0.174	80.011	1	0.0 00	0.21 2		

**Abbreviations:** 95%CI, 95% confidence interval; OR, odds ratio.

The multivariate analysis identified “the number of tendons > than 2” and “belonging to the critical group” as the primary factors associated with treatment failure. This means that when the multivariate analysis was performed, adjusting for all significant factors, the influence of other variables associated with failure in the univariate analysis was excluded.

The model exhibited significant coefficients and a substantial OR, with an accuracy of 72.0%, a sensitivity of 45.2%, and a specificity of 80.3%. In practical terms, this indicates that the proposed model correctly classified 72.0% of the sample, accurately identified 45.2% of the failure cases, and correctly recognized 80.3% of the non-failure cases.

## Discussion

Gasbarro et al. 4 showed that larger lesions significantly correlate with the failure of rotator cuff repairs compared to smaller or medium-sized lesions. Similarly, Wu et al. 2 emphasized that lesion size is a significant predictor of re-rupture among various intra-operative factors. Le et al. 3 found a correlation between lesion size, muscle quality, and early tendon re-rupture (up to 6 months postprocedure).

In our study, lesion size and the number of affected tendons were critical factors influencing outcomes. The failure rate among patients with up to 2 affected tendons was 17.8%, increasing to 37.5% for those with more than 2 affected tendons, with an OR of 2.8. This aligns with existing literature. Additionally, our study confirmed that lesions larger than 25 mm presented a higher OR for poor prognosis, especially when associated with more than 2 affected tendons. The risk of surgical failure for patients with more than 2 affected tendons and lesions larger than 25 mm was 2.89 times higher than for those outside this group.

Kim et al. 12 and our analysis both demonstrated that lesion sizes with more than 2.2 cm of retraction and muscle atrophy are highly predictive of non-healing repairs. A prolonged wait for suture correlates with increased failure risk due to lesion size progression and tendon atrophy. Our study found significant distinctions in the Goutallier classification of the infraspinatus muscle, especially in patients aged 75 or older, with higher fatty degeneration levels. This was less pronounced in younger age groups.

According to Yoon et al. 17 and Zhao et al. 18 infraspinatus infiltration with more than grade 2 fatty degeneration is directly related to re-rupture, with a sensitivity of 98% and specificity of 83.6%. This characteristic was prominent in the group aged over 75 years in our study. We concluded that patients up to 75 years old do not show age as an isolated factor of poor prognosis and are suitable candidates for surgical treatment to maintain better upper limb functionality.

In 2.6% of our sample, we identified a critical group based on the combination of age (over 75 years), muscle degeneration (Goutallier classification of the infraspinatus muscle greater than 2), and lesion diameter (greater than 25 mm). These patients had a high incidence of poor prognosis, with a treatment failure rate exceeding 75.0% and an OR of 11.2. We suggest that arthroscopic rotator cuff repair may not be the best option for these critical patients and that alternative treatments should be considered to enhance their quality of life and upper limb functionality.

This study has several limitations. Its retrospective nature introduces biases, such as selection and recall bias, affecting the generalizability of the findings. The absence of postoperative MRI data is significant, as MRI assesses the integrity of rotator cuff repairs and tendon healing. Without this data, our analysis relies solely on clinical and functional outcomes, which may not fully capture anatomical success. Future studies with postoperative MRI could provide a more comprehensive understanding and validate our findings. Furthermore, our sample was limited to patients treated at a single hospital by one surgical team, which may limit external validity. Despite rigorous statistical methods, unmeasured confounding variables may still influence outcomes, so caution is needed when interpreting the results.

## Conclusion

Rotator cuff arthroscopic repair did not show a worse prognosis for patients based on age alone up to the age of 75 years. However, the critical group identified (age > 75 years, Goutallier classification > 2 for the infraspinatus muscle, and lesion size > 25 mm) had significantly increased chances of suboptimal functional outcomes following rotator cuff repair.
