# Supplementary material for: Critical Predictors of the Outcomes of Rotator Cuff Arthroscopic Repair
Source: Rev Bras Ortop (Sao Paulo). 2025 Apr 28;60(1):s00451802963. doi: 10.1055/s-0045-1802963 (PMC12037216; doi:10.1055/s-0045-1802963)
Supplement: Related texts

# Research article: Principais fatores preditivos dos desfechos do reparo artroscópico do manguito rotador

**Keywords:** artroscopia, dor de ombro, estudos retrospectivos, idoso, lesões do manguito rotador

## Abstract

**Objetivo** Avaliar os desfechos funcionais após o reparo artroscópico do manguito rotador em uma população adulta e estabelecer os fatores de risco para o prognóstico ruim deste procedimento.

**Métodos** Este estudo de coorte retrospectiva analisou os prontuários médicos de 302 ombros submetidos à artroscopia para reparo do manguito rotador entre 1° de outubro de 2014 e 31 de dezembro de 2017. Os pacientes foram incluídos se tivessem uma lesão completa do manguito rotador e atendessem a outros critérios específicos. Os procedimentos cirúrgicos foram realizados de forma consistente e o tratamento pós-operatório envolveu um protocolo de reabilitação estruturado. A principal medida de desfecho foi a necessidade de cirurgia adicional ou um escore de ombro da University of California, Los Angeles (UCLA) < 28 em 2 anos após a cirurgia. As análises estatísticas incluíram métodos descritivos, inferenciais e de regressão logística.

**Resultados** O sucesso do tratamento foi observado em 234 ombros (77,5%), enquanto 68 ombros (22,5%) foram classificados como falhas de tratamento. Fatores significativamente associados à falha do tratamento incluíram o acometimento de mais de 2 tendões (razão de chances [RC]: 2,8; intervalo de confiança de 95% [IC95%]: 1,5–5,0) e lesão com mais de 25 mm de tamanho (RC: 2,9; IC95%: 1,6–5,3). Pacientes com 75 anos ou mais apresentaram taxa de falha de 54,5% em comparação a 21,3% em pacientes mais jovens ( *p* = 0,019). A classificação de Goutallier de degeneração gordurosa foi significativamente pior em pacientes mais velhos ( *p* = 0,001). Pacientes críticos, definidos como aqueles com idade ≥ 75 anos, classificação de Goutallier > 2 e tamanho de lesão > 25 mm, apresentaram taxa de falha do tratamento de 75,0% (RC: 11,2; IC95%: 2,2–57,0).

**Conclusão** A artroscopia do manguito rotador não mostrou pior prognóstico com base somente na idade até os 75 anos. No entanto, nos pacientes com mais de 75 anos, degeneração muscular significativa e lesões grandes tiveram chances substancialmente maiores de desfechos ruins. Tratamentos alternativos devem ser considerados para melhorar a qualidade de vida e a funcionalidade dos membros superiores desses pacientes críticos.

## Introdução

O aumento da expectativa de vida e o desejo de manter condições saudáveis no mercado de trabalho levaram os ortopedistas a reconsiderarem o tratamento conservador de lesões completas do manguito rotador em adultos. Aproximadamente 50% dos pacientes com mais de 70 anos têm rupturas assintomáticas do manguito rotador de espessura total e cerca de metade dessas lesões se torna sintomática em 2,8 anos, com necessidade de reparo do tendão para recuperação da função normal. 1 Apesar da correlação estabelecida entre degeneração do tendão, tamanho da ruptura e idade do paciente com os desfechos pós-operatórios, dados mais abrangentes são necessários para orientar decisões de tratamento de forma eficaz.

As lesões do manguito rotador são uma causa comum de dor e incapacidade no ombro, principalmente entre os idosos. Estudos anteriores mostraram que fatores como tamanho da ruptura, degeneração do tendão e idade do paciente influenciam significativamente os desfechos das cirurgias de reparo do manguito rotador. 2 3 4 Gasbarro et al. 4 e Wu et al. 2 demonstraram que rupturas maiores e a maior degeneração do tendão estão associadas a maiores taxas de falha cirúrgica. Da mesma forma, a idade do paciente foi identificada como um fator crítico, já que pacientes mais velhos frequentemente apresentam desfechos piores devido à menor capacidade de cicatrização e maior número de comorbidades. 3

No entanto, não há consenso sobre o limite de idade específico além do qual o prognóstico piora de maneira significativa. Além disso, o efeito combinado da idade, tamanho da ruptura e degeneração do tendão sobre os desfechos funcionais não foi investigado por completo em uma coorte de grande porte. Essa lacuna ressalta a necessidade de mais pesquisas para identificação de fatores preditivos críticos de sucesso ou fracasso cirúrgico no reparo do manguito rotador.

O objetivo deste estudo foi avaliar o impacto da idade, da degeneração do tendão e do tamanho da ruptura nos desfechos funcionais após o reparo artroscópico do manguito rotador. Também determinamos um limite crítico de idade associado a um pior prognóstico e identificamos os principais fatores preditivos de falha do tratamento nessa população. Ao proporcionar uma compreensão diferenciada desses fatores, esperamos auxiliar os cirurgiões a tomar decisões mais informadas sobre a adequação do reparo do manguito rotador em seus pacientes.

## Materiais e Métodos

## Delineamento do Estudo

Este estudo de coorte retrospectivo seguiu as diretrizes Strengthening the Reporting of Observational studies in Epidemiology (STROBE) 5 e a Declaração de Helsinque. Analisamos nossos registros médicos hospitalares de todas as cirurgias consecutivas de artroscopia do ombro para reparo de lesões completas do manguito rotador realizadas entre 1° de outubro de 2014 e 31 de dezembro de 2017, resultando em uma amostra elegível de 302 ombros.

Os critérios de inclusão foram todos os pacientes com lesões do manguito rotador admitidos para cirurgia artroscópica para reparo do tendão do manguito rotador. Os critérios de exclusão foram pacientes menores de 18 anos, aqueles com avaliações clínicas ou de imagens indisponíveis em prontuários médicos, já submetidos a cirurgia prévia no ombro, com lesões irreparáveis no manguito e com o ombro contralateral já incluído.

## Procedimentos Cirúrgicos

Todas as cirurgias foram realizadas em um único hospital pelos mesmos dois cirurgiões de ombro. Os pacientes foram posicionados em decúbito lateral com o braço mantido por um dispositivo de tração em 15 graus de flexão para frente e 45 graus de abdução com até 3 kg de tração.

Três portais (posterior, anterior e lateral) foram usados para inspecionar o labrum, o tendão do bíceps e os tendões do manguito rotador. As lesões foram reparadas com uma a quatro âncoras de sutura absorvíveis até o fechamento adequado. A técnica cirúrgica incluiu acromioplastia e, se necessário, tenotomia ou tenodese do bíceps. Técnicas específicas de reparo, como suturas de fileira única ou dupla, foram empregadas com base na qualidade do tendão e no tamanho da ruptura.

## Cuidado Pós-operatório

O tratamento pós-operatório envolveu o uso de tipoia e almofadas de abdução por 6 semanas.

Os exercícios de fortalecimento começaram 12 semanas após a cirurgia. Os pacientes foram avaliados usando exames clínicos e escalas funcionais em intervalos regulares, sendo a última avaliação 2 anos após a cirurgia.

## Medidas de Desfechos

O desfecho primário foi a necessidade de cirurgia adicional ou um escore de ombro da University of California, Los Angeles (UCLA) < 28 2 anos após a cirurgia, indicando falha do tratamento. 6 A degeneração dos músculos do manguito foi avaliada por ressonância magnética e classificada segundo Goutallier. 7 8 As lesões foram medidas durante a cirurgia com uma sonda de 5 mm e categorizadas como menores que 25 mm ou iguais ou superiores a 25 mm. 9

## Análise Estatística

Realizamos análises descritivas e inferenciais para determinar o perfil dos pacientes em 2 grupos com base no prognóstico (com e sem falha de tratamento) e no escore de ombro da UCLA antes e 2 anos após a cirurgia. A variabilidade na distribuição das variáveis quantitativas foi considerada baixa para o coeficiente de variação (CV) < 0,20, moderada para 0,20 ≤ CV < 0,40 e alta para CV ≥ 0,40. A análise estatística inferencial avaliou a significância das diferenças em grupos independentes, escore de ombro da UCLA pré e pós-operatório e correlações variáveis.

A avaliação das correlações entre duas variáveis qualitativas utilizou o teste do qui-quadrado ou Fisher. Caso houvesse correlação significativa, a razão de chances (RC) estimou o risco de falha do tratamento segundo o intervalo de confiança de 95% (IC95%).

A análise inferencial de variáveis quantitativas comparou as distribuições de variáveis quantitativas ou ordinais em dois grupos independentes usando o teste de Mann-Whitney. Para comparações com mais de dois grupos independentes, o teste de Kruskal-Wallis foi usado. O teste de Wilcoxon comparou o escore de ombro da UCLA antes e depois da artroscopia.

No teste de postos sinalizados de Wilcoxon, o efeito da intervenção recebida no escore de ombro da UCLA foi avaliado usando a medida do tamanho do efeito Δ de Glass: Δ, em que *m*_1_ indica a média da variável na primeira avaliação (pré-intervenção), *m*_2_ é a média da variável da segunda avaliação (pós-intervenção) e *s*_1_ é o desvio-padrão da variável antes da intervenção. Este índice é utilizado em ensaios clínicos 7 e compreende o valor amostral da diferença média padronizada por meio do desvio-padrão do grupo controle sem falha, que reflete melhor o desvio-padrão da população. 10 11 12

A classificação do Δ de Glass proposta por Rosenthal ^10^ foi utilizada da seguinte forma: *Δ* ≤ 019, efeito insignificante; 020 ≤ *Δ* ≤ 049, efeito pequeno; 050 ≤ *Δ* ≤ 079, efeito médio; 080 ≤ *Δ* ≤ 129, efeito grade; e *Δ* ≥ 130, efeito muito grande. O tamanho do efeito segundo os testes não paramétricos de Wilcoxon ( *r* ) também foi calculado pelo quociente entre o valor z (do teste de Mann-Whitney) e a raiz quadrada do número total da amostra. A classificação adotada para o tamanho do efeito dos testes não paramétricos foi a proposta por Pallant: *r* < 01–muito pequeno; 01 < *r* ≤ 03–pequeno; 03 < *r* < 05–médio; e *r* > 05–ótimo. 13

A associação entre duas variáveis quantitativas foi investigada segundo o coeficiente de correlação de postos de Spearman. A correlação foi considerada forte se o valor absoluto fosse > 0,7 e moderada se 0,65 > 0,7. O método de Wald, ou *forward stepwise technique* , foi usado para seleção de variáveis na análise de regressão logística com ponto de corte igual à proporção de falhas na amostra. A qualidade do modelo foi avaliada pela significância dos parâmetros do modelo final, RC e medidas de precisão, sensibilidade e especificidade. Todos os testes de significância foram conduzidos com um nível de significância máximo de 5% ( *p* < 0,05), rejeitando a hipótese nula se o valor de *p* fosse menor que 0,05. 11 14 15 16

## Resultados

O estudo inicialmente incluiu 332 ombros. Com base nos critérios de exclusão, 6 ombros com lesões irreparáveis identificadas durante a cirurgia, 5 ombros com dados médicos insuficientes e 19 ombros contralaterais de pacientes já incluídos foram removidos. Consequentemente, os resultados são baseados em uma amostra elegível de 302 ombros que foram submetidos à artroscopia para reparo do manguito rotador.

## Características dos Procedimentos Cirúrgicos

Em resumo, os pacientes submetidos à artroscopia para sutura do tendão geralmente apresentavam lesões do lado direito (72,5%). A maioria das lesões era degenerativa (93,4%) e a técnica de “fileira única” foi usada em 92,1% dos casos. As lesões predominantemente tinham menos de 25 mm de tamanho (56,6%) e dois tendões foram mais comumente acometidos (49,7%). Em 52,0% dos casos, não houve alterações na cabeça longa do bíceps e a tenodese do bíceps foi realizada em 37,7% dos pacientes. O tipo mais comum de acrômio foi o tipo II (49,7%) e a incidência de instabilidade associada à ruptura do tendão foi de 20%.

## Desfechos do Tratamento

O sucesso do tratamento foi observado em 234 ombros (77,5%), enquanto 68 ombros (22,5%) foram classificados como falhas de tratamento. A falha do tratamento foi definida pela necessidade de cirurgia adicional ou um escore de ombro da UCLA < 28 2 anos após a cirurgia. Entre as falhas, 67 casos (22,2%) foram devido ao escore de ombro da UCLA indicando função ruim e 15 casos exigiram cirurgia adicional ( Fig. 1 ).

**Fig. 1:** Distribuição dos casos por desfechos observados 2 anos após a artroscopia.

O teste de Kruskal-Wallis não mostrou diferença significativa entre os grupos no escore de ombro da UCLA antes e após a cirurgia, nem na melhora entre pacientes com cabeça longa do bíceps intacta ou aqueles submetidos à tenodese ou tenotomia.

A melhora no escore de ombro da UCLA não foi correlacionada ao tipo de acrômio ou ângulo crítico do ombro, indicando que essas variáveis não afetaram os desfechos de forma significativa.

## Influência do Acometimento de Tendões

Ao analisar a frequência das características das lesões dos pacientes da amostra, observou-se que a distribuição do número de tendões acometidos foi significativamente diferente nos grupos com e sem falha de tratamento ( *p* = 0,009). Ao dicotomizarmos essa variável em “mais de dois tendões acometidos” (sim ou não), ficou evidente a associação com o pior prognóstico entre aqueles que se enquadraram nessa classificação (17,8% *versus* 37,5%; *p* < 0,001). A RC foi de 2,8 (IC95%; 1,5–5,0).

## Tamanho da Lesão

As lesões foram categorizadas como < 25 mm ou ≥ 25 mm.

Em pacientes com lesões ≥ 25 mm, a RC para falha do tratamento foi de 2,9 (IC95%: 1,6–5,3), indicando um aumento significativo nos desfechos ruins.

Também examinamos o efeito do tamanho da lesão e a presença de mais de dois tendões acometidos no prognóstico. Nossos achados mostraram que 23,2% dos casos tinham mais de dois tendões acometidos e lesões ≥ 25 mm. Dentro do grupo com prognóstico favorável, apenas 18,4% tinham essas características combinadas. Por outro lado, no grupo de prognóstico ruim, 39,7% tinham mais de 2 tendões acometidos e lesões ≥ 25 mm ( *p* < 0,001). A RC para prognóstico ruim em pacientes com esses fatores combinados foi de 2,9, indicando que esses pacientes tinham quase 3 vezes mais probabilidade de apresentar desfechos ruins. Essa RC foi significativa, com IC de 1,6 a 5,3, o que significa que o resultado é estatisticamente robusto e o IC não inclui 1.

## Idade como Fator Preditivo

A idade previu significativamente os desfechos do tratamento. Pacientes com menos de 75 anos tiveram uma taxa de falha de 21,3%, enquanto aqueles com 75 anos ou mais tiveram uma taxa de falha de 54,5% ( *p* = 0,019). No entanto, não houve diferença significativa na melhora do escore de ombro da UCLA entre essas faixas etárias ( *p* = 0,068).

## Classificação de Goutallier

A classificação de Goutallier de degeneração gordurosa do músculo infraespinhoso foi significativamente pior em pacientes com 75 anos ou mais em comparação a pacientes mais jovens ( *p* = 0,001). Esta classificação avaliou o grau de degeneração muscular e seu impacto nos desfechos cirúrgicos ( Tabela 1 e Fig. 2 ).

**Fig. 2:** Diagramas de caixa das distribuições da classificação de Goutallier do infraespinhoso de acordo com a faixa etária.

**Tabela 1:** Principais estatísticas não paramétricas da classificação de Goutallier nos subgrupos definidos de acordo com a faixa etária para os pontos de corte de 70 e 75 anos

Classificação de Goutallier	Idade < 70 anos ( *n* = 270)	Idade ≥ 70 e < 75 anos ( *n* = 21)	Idade ≥ 75 anos ( *n* = 11)	Valor de *p* no teste de Kruskal-Wallis
SUPRAESPINHOSO				
Mínimo	0,0	0,0	1,0	**0,007**
Máximo	4,0	4,0	4,0	
Quartil 1	**1,0**	**1,0**	**2,0**	
Mediana	2,0	2,0	3,0	
Quartil 3	3,0	3,0	3,0	
**INFRAESPINHOSO**				**0,001**
Mínimo	0,0	0,0	0,0	
Máximo	4,0	3,0	4,0	
Quartil 1	0,0	1,0	2,0	
Mediana	1,0	1,0	2,0	
Quartil 3	1,0	2,0	2,5	
**SUBESCAPULAR**				0,189
Mínimo	0,0	0,0	0,0	
Máximo	3,0	3,0	2,0	
Quartil 1	**0,0**	**0,0**	**0,5**	
Mediana	1,0	1,0	1,0	
Quartil 3	1,0	1,0	1,0	

## Análise do Grupo Crítico

Os pacientes críticos, definidos como aqueles com idade ≥ 75 anos, classificação de Goutallier do músculo infraespinhoso acima de 2 e lesão ≥ 25 mm, representaram 2,6% da amostra (8 indivíduos). A incidência de prognóstico ruim nesse grupo foi de 75,0% em comparação a 21,1% em pacientes não críticos ( *p* < 0,001), como mostra a Fig. 3 . A RC para falha do tratamento no grupo crítico foi de 11,2 (IC95%: 2,2–57,0).

**Fig. 3:** Diagramas de caixa das medidas do escore de ombro UCLA antes e depois da cirurgia no grupo crítico e no grupo não crítico.

## Análise de Regressão Logística

Todos os fatores significativos identificados foram propostos como preditivos de falha em uma análise de regressão logística multivariada. A Tabela 2 mostra os resultados do grupo crítico. O método de Wald foi empregado para seleção das variáveis, utilizando um ponto de corte igual à proporção de falhas de tratamento na amostra (0,23). Os resultados do modelo final são apresentados na Tabela 3 .

**Tabela 2:** Análise dos fatores associados à falha

Fator	Falha				Valor de *p* *	RC (IC95%)
	Não		Sim			
**Grupo crítico = idade > 75 anos + IE com Goutallier > 2 + lesão > 2,5 cm**						
Não	232	78,9%	62	21,1%	< 0,001**	**11,2 (2,2–57,0)**
Sim	2	25,0%	6	75,0%		

**Abreviaturas:** IC95%, intervalo de confiança de 95%; IE, infraespinhoso; RC, razão de chances.

**Notas:** *Teste do qui-quadrado. **Teste exato de Fisher.

**Tabela 3:** Modelo de regressão logística para ocorrência de falha

Fator	Coeficiente B	Erro padrão	Dados de Wald	Grau de liberdade	Valor de *p*	RC = Exp (B)	Limite inferior do IC95% para Exp (B)	Limite superior do IC95% para Exp (B)
Número de tendões > 2	1,075	0,296	13,193	1	0,000	2,929	1,640	5,230
Pertencer ao grupo crítico	1,896	0,854	4,926	1	0,026	6,660	1,24 8	35,531
Constante	-1,553	0,174	80,011	1	0,000	0,212		

**Abreviaturas:** IC95%, intervalo de confiança de 95%; RC, razão de chances.

A análise multivariada identificou “o número de tendões > dois” e “pertencer ao grupo crítico” como os principais fatores associados à falha do tratamento. Isso significa que, à análise multivariada, ajustando para todos os fatores significativos, a influência de outras variáveis associadas à falha na análise univariada foi excluída.

O modelo exibiu coeficientes significativos e uma RC substancial, com precisão de 72,0%, sensibilidade de 45,2% e especificidade de 80,3%. Em termos práticos, isso indica que o modelo proposto classificou corretamente 72,0% da amostra, identificou com precisão 45,2% dos casos de falha de tratamento e reconheceu corretamente 80,3% dos casos sem falha.

## Discussão

Gasbarro et al. 4 mostraram que lesões maiores são correlacionadas de forma significativa à falha dos reparos do manguito rotador em comparação a lesões menores ou médias. Da mesma forma, Wu et al. 2 enfatizaram que o tamanho da lesão é um fator preditivo significativo de nova ruptura entre vários fatores intraoperatórios. Le et al. 3 observaram uma correlação entre o tamanho da lesão, a qualidade do músculo e a nova ruptura precoce do tendão (até 6 meses após o procedimento).

Em nosso estudo, o tamanho da lesão e o número de tendões acometidos foram fatores críticos que influenciaram os desfechos. A taxa de falha de tratamento entre pacientes com até 2 tendões acometidos foi de 17,8%, aumentando para 37,5% naqueles com mais de 2 tendões acometidos, com RC de 2,8. Isso condiz com a literatura existente. Além disso, nosso estudo confirmou que lesões maiores que 25 mm apresentaram OR maior para prognóstico ruim, especialmente quando associadas ao acometimento de mais de 2 tendões. O risco de falha cirúrgica em pacientes com mais de 2 tendões acometidos e lesões > 25 mm foi 2,89 vezes maior nos demais indivíduos.

A análise de Kim et al. 12 e a nossa demonstraram que lesões com mais de 2,2 cm de retração e atrofia muscular são altamente preditivos de reparos não cicatrizantes. Uma espera prolongada pela sutura se correlaciona ao aumento do risco de falha devido à progressão do tamanho da lesão e atrofia do tendão. Nosso estudo observou distinções significativas na classificação de Goutallier do músculo infraespinhoso, especialmente em pacientes com 75 anos ou mais, com níveis maiores de degeneração gordurosa. Isso foi menos pronunciado em faixas etárias mais jovens.

De acordo com Yoon et al. 17 e Zhao et al., 18 a infiltração do infraespinhoso por degeneração gordurosa em grau > 2 está diretamente relacionada à nova ruptura, com sensibilidade de 98% e especificidade de 83,6%. Essa característica foi proeminente no grupo com mais de 75 anos em nosso estudo. Concluímos que pacientes com até 75 anos não apresentam a idade como fator isolado de prognóstico ruim e são candidatos adequados ao tratamento cirúrgico para manter melhor funcionalidade do membro superior.

Em 2,6% da nossa amostra, identificamos um grupo crítico com base na combinação de idade (> 75 anos), degeneração muscular (classificação de Goutallier do músculo infraespinhoso > 2) e diâmetro da lesão (> 25 mm). Esses pacientes tiveram alta incidência de prognóstico ruim, com taxa de falha do tratamento superior a 75,0% e RC de 11,2. Sugerimos que o reparo artroscópico do manguito rotador pode não ser a melhor opção para esses pacientes críticos e que tratamentos alternativos devem ser considerados para melhorar sua qualidade de vida e funcionalidade do membro superior.

Este estudo teve várias limitações. Sua natureza retrospectiva introduziu vieses, como viés de seleção e de memória, afetando a generalização dos achados. A ausência de dados de ressonância magnética pós-operatória é significativa, pois este exame avalia a integridade dos reparos do manguito rotador e a cicatrização do tendão. Sem esses dados, nossa análise depende apenas de desfechos clínicos e funcionais, que podem não representar por completo o sucesso anatômico. Estudos futuros com ressonância magnética pós-operatória podem fornecer uma compreensão mais abrangente e validar nossos achados. Além disso, nossa amostra foi composta apenas por pacientes tratados em um único hospital por uma equipe cirúrgica, o que pode limitar a validade externa. Apesar dos métodos estatísticos rigorosos, variáveis de confusão ainda não medidas podem influenciar os desfechos; logo, é necessário cautela ao interpretar os resultados.

## Conclusão

O reparo artroscópico do manguito rotador não teve prognóstico ruim em pacientes com base apenas na idade até os 75 anos. No entanto, o grupo crítico identificado (idade > 75 anos, classificação de Goutallier > 2 no músculo infraespinhoso e tamanho da lesão > 25 mm) teve chances significativamente maiores de desfechos funcionais subótimos após o reparo do manguito rotador.
